# Autopsy findings and pattern of mortality in Nigerian sickle cell disease patients

**DOI:** 10.11604/pamj.2014.18.30.4043

**Published:** 2014-05-08

**Authors:** Gabriel Olabiyi Ogun, Henry Ebili, Taiwo Racheal Kotila

**Affiliations:** 1 Department of Pathology, College of Medicine, University of Ibadan, University College Hospital, Ibadan, Nigeria; 2 Department of Hematology, College of Medicine, University of Ibadan, University College Hospital, Ibadan, Nigeria

**Keywords:** Sickle cell disease, cause of death, mortality, autopsy

## Abstract

**Introduction:**

Sickle Cell Disease (SCD) has a high mortality rate in the environment where we practice. There is lack of contemporal autopsy studies describing causes of death among SCD patients at our centre.

**Methods:**

This is a retrospective study of SCD patients who died between January 1991 and December 2008 and that had autopsy examination to confirm the cause of death in a Nigerian teaching hospital. The clinical data, including the age, gender, Hb genotype, and the major autopsy findings and cause of death were obtained for each patient from the complete autopsy reports that included histopathological examination. Multiple causes of death were entertained.

**Results:**

A total of 52 autopsies were performed. The mean age at death was 21.3 years (range, 1-47 years) and a male/female ratio of 1.3:1. HbS+C patients lived longer than HbS patients (21.0 years Vs 24.0 years) and peak mortality was in the 2nd and 3rd decades of life. The commonest causes of death as a single entity or in combination included infections in 78% of cases, fatal thrombotic/embolic events (37%) making acute chest syndrome a leading cause of death. This was followed closely by anemia alone or in combination with acute sequestration crises in 31% of patients.

**Conclusion:**

Infections are the commonest causes of death in Nigerian SCD patients, efforts to reduce infection especially early in life through prophylaxis or vaccination will impact on the overall survival of these patients.

## Introduction

Sickle cell disease (SCD) describes the conditions in which the haemoglobin (Hb)S gene is inherited with another abnormal β chain gene, which include the homozygote state (HbSS) and the double heterozygote state (HbSC or HbS- β thalassaemia). The homozygote state also known as sickle cell anemia (SCA) is the commonest variant and has the severest clinical manifestations of any of the SCD variants. The heterozygote state (sickle cell trait or HbAS) is usually normal phenotypically. Sickle cell disease is the commonest haemoglobinopathy and single gene disorder in black Africans. In the Nigerian population, carrier rate for the trait (HbAS) is about 25% while 2-3% suffers from SCA [1]. Diagnosis of SCD in Nigeria is by haemoglobin electrophoresis at alkaline pH alone, so patients are either diagnosed as HbS or HbS+C and a report is stuck on the case file of the patients. Such reports may however be missing in some patients if the case file becomes voluminous and a new file is opened. Autopsies are performed at the request of the Haematologist after the demise of a patient in the hospital after obtaining consent from the relatives and rarely when a patient is brought in dead after having died at home or while in transit to the hospital. We sought to investigate the pattern and causes of mortality in SCD at our centre, with emphasis on the causes of death among SCD patients at our hospital. We believe that the findings of the study would help direct clinical management to focus attention on these specific causes of death, which with good management and policy may improve morbidity and mortality. This will be by the way of aiding clinical preparedness, streamlining patient counseling and enabling preventive therapies. In addition, it will add to the existing epidemiological data, knowledge of the disease and possibly the pathogenesis of the disease.

## Methods

This is a retrospective study of SCD patients who died between January 1991 and December 2008 and had autopsy examination to confirm the cause of death at the Department of Pathology, University College Hospital, Ibadan. The clinical history/data, including the age, gender, Hb genotype, and the major autopsy findings and cause of death were obtained for each patient from the complete autopsy reports that included histopathological examination. Patients were included in the review if they were referred from the Haematology department as having SCD and this was corroborated by the report of the electrophoresis in the case file. The immediate and specific (diseases or conditions directly leading to death) cause of death were recorded according to the internationally accepted/ World Health Organization (WHO) medical certification of cause of death. Multiple or joint causes of death was entertained. A broad classification of the cause of death under the following rubrics was done- infections, thromboembolism, Severe Anaemia with Cardiac failure, Acute Hepatospleenic sequestration, Aspiration pneumonitis, Intracranial haemorrhage and Massive Erosive gastritis and duodenitis. This study was conducted in compliance with the guidelines of the Helsinki declaration on biomedical research in human subjects. Confidentiality of the identity of the patients and personal health information was maintained.

## Results

**Figure 1:**
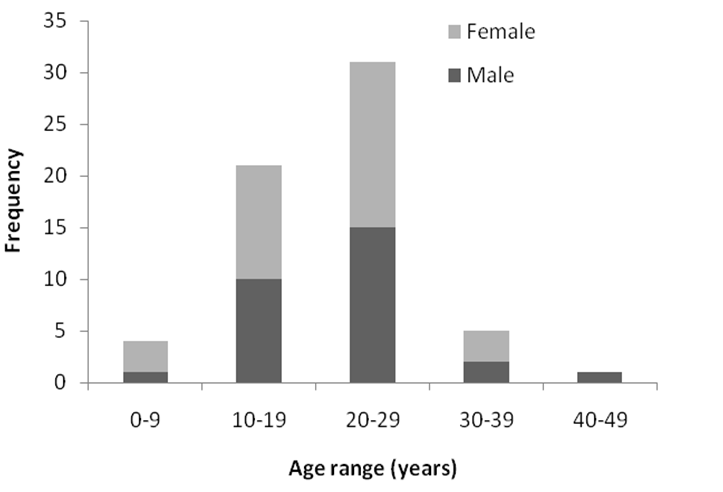
Age group and gender distribution of patients

A total of 52 cases were seen in the seventeen year period out of which 48 had electrophoresis report in the clinical notes but the report was missing in four. Forty (83.3%) had SCA while 8(16.6%) were Hb S+C. The mean age was 21.0 years with a range of 1-47 years. HbS+C and male patients were slightly older 24 Vs 21 years and 22.7 Vs 19.4 years respectively. Peak mortality occured in the 2nd and 3rd decades of life but with equal sex distribution, more deaths occurred however in the female child in the first decade of life (Figure 1). Infections and thromboembolic phenomenon were the major cause of death, contributing to demise in 78% and 37% of cases respectively (Table 1). This was followed by severe anemia contributed as cause of death in about 31% of cases, but deaths arising from anaemia was caused by sequestration into either or both the liver or spleen in a third of such patients (Table 1). Infections of the respiratory system affected more than half of the patients (53.8%, Table 2), thromboembolic phenomenon also affected the respiratory system in half of the patients (Table 3) making acute chest syndrome the leading cause of death.

**Table 1:** Broad classification of the causes of death

Cause of Death	Number of Cases*	%
Infections	41	78
Thromboembolism	18	37
Severe Anaemia	11	21
Hepatospleenic Sequestration (with consequent Severe anaemia)	5	9.6
Aspiration pneumonitis	4	7.6
Intracranial Haemorrhage	2	3.8
Erosive gastritis and duodenitis	2	3.8

* The number of cases and percentages are more than 100% because there were more than single plausible causes of death in most patients

**Table 2:** Systemic localization of infections causing death

Infections	Number of Cases	Percentage
Respiratory	22	53.8
Cerebral	7	17.1
Urinary Tract	7	17.1
Disseminated Tuberculosis	2	4.8
Endocarditis	1	2.4
Peritonitis	1	2.4
Cholecystitis	1	2.4
**Total**	**41**	**100**

**Table 3:** Organ/system location of thrombo-embolic phenomena

Organ/system	Number of cases	Percentage
Pulmonary	9	50%
Renal	3	16.6%
Mesenteric	2	11.1%
Cardiac	1	5.6%
Hepatic	1	5.6%
Cerebral	2	11.1%
**Total**	**18**	**100%**

## Discussion

The finding of 52 deaths in a 17 year period does not translate to a low death rate in the Nigerian SCD patients, since the recorded low death rate may be due to the fact that family members often do not consent to autopsy because of religious and social reasons [2]. Secondly, not all deaths are reported, especially when it occurs in the home setting and for a disease condition that is emotionally and financially tasking for the family. This is especially so in early childhood when children will succumb to the illness as a result of infection or anaemia before getting to the hospital.

The mean age at death of 21 years is similar to the mean age of 22 years obtained from a clinical review of sickle cell disease patients about the same time in our centre [3]. This would suggest that the median survival of the Nigerian SCD patient is much lower than that of Jamaican or USA patients [4]. The mean age at death for HbSS and HbSC, of our patients is comparable to the findings of Manci et al. [5] who found a mean age at death of 20.7 years among HbSS patients and 25 years among HbSC patients from across several centres in the United States. However, this mean age at demise are much lower than what has been reported in other series in the United States [6, 7], Jamaica [8], France and England [9] where the age at death were between 32 years and 45 years for SCA and up to 68 years for HbS+C. The higher survival in those studies could be attributed to easy access of SCD patients to health care and health education in the locales where these studies were done. Infection and thromboembolic events are leading cause of death in SCD patients as shown by other studies [6–10]. These were categorized as acute chest syndrome as done by Thomas et al and Platt et al. [6, 8]. The study from England and France however reported vaso-occlusion related causes as the most common cause of death but this was followed closely by infection [9]. Meningitis featured prominently as an infectious cause of death in this study, being responsible for 13.2% (7 out of 53) cases of deaths among our patients. Meningitis was similarly a major cause of death in other series by Thomas et al and Perronne et al where it was the commonest infective cause of death [8, 9].

SCD patients are more prone to infections because of autosplenectomy that occurs as a result of repeated infarction of the organ thus leading to the loss and inability to fight infection. Additionally, SCD patients have defective alternative pathway of complement activation and reduced ability of neutrophils to kill pathogenic organisms which makes them especially prone to infection by encapsulated organisms. Common bacterial organisms that have been documented to cause infection in SCD patients in our environment include Salmonella typhi, E. coli, Staphylococcus Aureus, Haemophillus influenza and Klebsiella species [11, 12], while Infection caused by Streptococcus pneumonia which have been reported by others have not been reported among SCD patients in our locale [11–13]. The high prevalence of infections by Haemophilus Influenza and Pneumococcus species in patients with SCD, is the reason why patients in the UK and USA are placed on prophylaxis and vaccinated against these organisms. The introduction of bacterial prophylaxis for SCD patients in Nigeria may therefore reduce the mortality rate especially in early childhood due to infections.

Another important cause of death in our cohort is severe anaemia associated with cardiac failure, playing a significant role as cause of death in 21% of cases. Cardiac failure was also an important mechanism of death in other studies [5–7, 9]. Other causes of cardiac failure in SCD patients may include myocardial infarction, arrhythmias, and cardiomyopathy [7] but these did not feature in our patients possibly because they do not live long enough to experience such complications. Organ sequestration, a spontaneous pooling of blood in solid and spongy viscera occur most commonly in the spleen, but also in the liver and lungs in SCD patients. Its pathogenesis is unknown but it usually follows or occurs concomitantly with acute viral infections, pain crisis, bacteraemia and stroke. It causes cardiovascular collapse and it is not an uncommon cause of death in this group of patients. In this study hepatosplenic sequestration was a cause of death in 9.6% of our patients, being the 4th commonest cause of death. Thomas et al and Pafrey et al found organ sequestration to be the second commonest cause of death (12% in both studies) [8, 10].

## Conclusion

This study has illustrated that the commonest cause of death in the SCD patients in the Nigerian SCD patient is acute chest syndrome which may present either as infections or thromboembolism in the respiratory system. The use of bacterial prophylaxis early in childhood is expected to reduce early mortality and thus improve the life expectancy of these patients.
